# Suppression of Strong Background Interference on E-Nose Sensors in an Open Country Environment

**DOI:** 10.3390/s16020233

**Published:** 2016-02-16

**Authors:** Fengchun Tian, Jian Zhang, Simon X. Yang, Zhenzhen Zhao, Zhifang Liang, Yan Liu, Di Wang

**Affiliations:** 1 College of Communication Engineering, Chongqing University, 174 Sha Pingba, Chongqing 400044, China; zj_cqu_2007@163.com (J.Z.); zhenzhen715@163.com (Z.Z.); liangzhifang0508@163.com (Z.L.); 20141202032@cqu.edu.cn (Y.L.); diwangcqu@163.com (D.W.); 2 Advanced Robotics and Intelligent Systems (ARIS) Lab, School of Engineering, University of Guelph, Guelph, ON N1G 2W1, Canada; syang@uoguelph.ca

**Keywords:** electronic nose sensors, outdoor, background interference, suppression

## Abstract

The feature extraction technique for an electronic nose (e-nose) applied in tobacco smell detection in an open country/outdoor environment with periodic background strong interference is studied in this paper. Principal component analysis (PCA), Independent component analysis (ICA), re-filtering and *a priori* knowledge are combined to separate and suppress background interference on the e-nose. By the coefficient of multiple correlation (CMC), it can be verified that a better separation of environmental temperature, humidity, and atmospheric pressure variation related background interference factors can be obtained with ICA. By re-filtering according to the on-site interference characteristics a composite smell curve was obtained which is more related to true smell information based on the tobacco curer’s experience.

## 1. Introduction

Electronic nose (e-nose) techniques are being more and more widely used in areas such as food evaluation, medical diagnosis, environmental monitoring and industrial control [1], but the interference from environmental temperature, humidity, atmospheric pressure variation and other non-target smells is still a bottleneck which frustrates people and limits the development of e-nose technology. Two factors cause this situation. On the one hand, the key part of the e-nose, *i.e*., most metal oxide semiconductor (MOS) gas sensors, are not perfect and their responses are easily affected by temperature, humidity and atmospheric pressure variations. On the other hand, the e-nose sensors are inherently susceptible to interference since each gas sensor of the e-nose should have cross sensitivity, *i.e*., it should respond to more than one gas. This is either an advantage or a disadvantage. The benefit of cross sensitivity is that an e-nose may measure many kinds of smells with a limited number of sensors, while various interferences will result in responses in the e-nose sensors. The interferences resulting from other gases and environmental factors cannot be effectively separated either by traditional filtering or by wavelet transforms since these interferences are almost entirely mixed with the target gases, which are to be detected, both in the frequency and time domain, so solving the problem of environmental interference suppression in an e-nose is an important task.

Currently the main methods of compensating for environmental temperature and humidity variations are methods based on temperature and humidity compensation models, artificial neural networks (ANNs), PCA combined with ANNs, *etc*. For example, a compensation method based on knowledge modification was proposed in [2], where the environmental temperature and humidity were used as inputs of an ANN to build the correction model. The ANN and the automatic Bayesian regularization were used to form an approximate regression function retaining good generalization properties to reduce the influence of the environment on an e-nose in [3]. In [4] the dimensions of sample data consisting of temperature, humidity and gas sensor responses were reduced by PCA, then features were chosen according to Wilks’ rule, and finally the compensation to ambient temperature and humidity was realized. A thermistor was adopted to compensate for the influence of temperature-incurred changes of MOS gas sensor responses in [5], while the effect of humidity was ignored. Two equal sensors were used to minimize the interference of temperature with one sensor for signal measurement and another as reference, the difference of the two sensors was used as the output signal in [6]. The responses of temperature and humidity sensors together with that of gas sensors were used as inputs of an ANN to realize compensation of temperature and humidity influences in [7,8]. The information of temperature and humidity sensors was input to a data merging center consisting of wavelet ANNs to perform compensation to environmental temperature and humidity changes in [9]. A physical way was used to compensate the temperature and humidity influence in [10], where SiO_2_ was used as the sensing material with a titanium thermistor being used to maintain a constant temperature. Combined with data standardization, PCA and ANN, the gas concentration prediction was realized with QCM as gas sensor [11]. Information of gas sensors under various temperature and humidity conditions was collected to calculate the corresponding drift coefficients, and a compensation was realized in [12]. After the sensor response signals were analyzed by ICA, the correlation of each independent component with temperature and humidity was calculated to determine and remove the temperature and humidity factors [13].

Meanwhile, the background interference correction of e-noses can be carried out by methods based on correlation, ICA, ANN as well as support vector machine (SVM). Instead of modifying the system hardware, these are software compensation methods. For example, to perform wavelet transform (WT) on sensor signals followed by calculating of spatial correlation coefficients of WT between smells of infected and healthy mice under the same WT scale, the background interference was minimized according to correlation coefficients [14]. Zhang *et al*. divided the anti-interference process into two steps: (1) determination of interference, (2) correction of the interfered signal. The gases were partitioned into two categories: target and non-target (*i.e*., those with the exception of the target). The response of each sensor was categorized first by least squared SVM (LSSVM), back propagation (BP) network and was replaced by the most nearby signal if it was judged as an interference [15]. Al-Maskari *et al*. adopted methods based on kernel Fuzzy C-Mean clustering and Fuzzy SVM to increase the sorting accuracy of e-nose data with noise and drift [16]. The sensor array output was analyzed by ICA with the responses to background interference being used to construct a reference vector, and the correlation coefficients between the components of ICA and the reference vector were calculated to determine and delete background interferences [17]. Feng *et al*. suppressed interference by removing the components of response matrix orthogonal to those of the target matrix with RFB parameters optimized by particle swarm optimization (PSO) [18].

In summary, various methods of environmental interference have been proposed, however, they were either used in the recognition/categorization of smells, or only suitable to the application scenarios where background interferences as only their temperature and humidity effects were compensated. We propose a method for effectively separating environment temperature, humidity, atmospheric pressure variation and reducing non-target smell interference. The remainder of the paper is arranged as follows: a brief overview of the tobacco curing process is introduced in Section 2. The developed system for collecting the smell, the analysis of the interference existing in the system and the proposed method to restrain the interference are presented in Section 3. The result of our method, *i.e*., the tobacco smell curve, is presented in Section 4. Then the data certification using CMC to validate the effectiveness of the proposed method and some discussions are provided in Section 5. Finally, conclusions are given in Section 6.

## 2. Tobacco Curing Process—A Brief Overview

**Figure 1:**
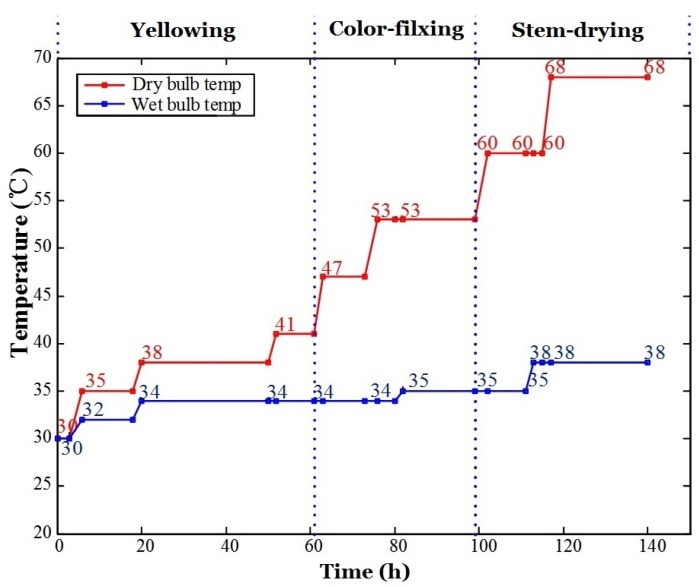
Three-stage-curing chart.

The curer’s chart for tobacco curing is illustrated in Figure 1. The three-stage-curing craft was adopted in our experiments, which is common in tobacco curing factories. The curing chart is divided into 19 stages in detail or three coarse stages (*i.e*., yellowing stage, color-fixing stage and stem-drying stage).

A flue-curing process may last six to seven days (144–168 h). The temperature and humidity in the curing barn are reflected by dry-bulb temperature and wet-bulb temperature (psychrometer), respectively. The red line in Figure 1 represents the dry-bulb temperature and the blue one denotes the wet-bulb temperature. The temperature and humidity changed during the whole curing process.

## 3. Experiments

### 3.1. Developed E-Nose System for Tobacco Flue-Curing

With the catalysis of various enzymes, thousands of chemical components are produced and they release their corresponding smells during tobacco curing [19]. Professionals may determine the curing stage and quality of tobacco by sniffing these smells. An e-nose may simulate the olfaction function of human beings, and it is expected to find out the features and rules of tobacco smell variation by utilizing the e-nose so as to provide a clue for automatic control of tobacco curing.

**Figure 2:**
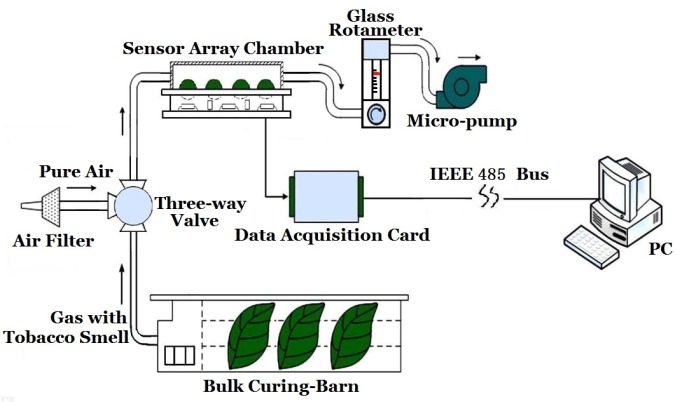
E-nose system designed for smell features extracting in tobacco curing.

Here low price and simplicity are the key points to be considered since this is a widely used application in tobacco factories, so a simple and specialized e-nose, rather than a commercialized common purpose e-nose for lab experiments, was adopted in our system. The specialized e-nose system designed for tobacco curing smell feature extraction is illustrated in Figure 2. The e-nose system comprises an air filter, sensor array, rotameter, vacuum micro-pump, data acquisition card and IEEE 485 bus, *etc*. The chemical components of tobacco which have important contributions to their smells can be mainly categorized as phenols, organic acids, lipid substances, aldehyde substances, alcohol substances, *etc*. [19,20], so a set of sensors including nine MOS gas sensors (*cf*., Table 1) and temperature, humidity, atmospheric pressure sensors were selected to detect smells during the tobacco flue-curing process.

Since ambient temperature, humidity and atmospheric pressure (T/H/P) have strong influences on the response of gas sensors, corresponding T/H/P sensors were used in the sensor array of the e-nose so as a compensation can be made in the subsequent algorithm. The outputs of the sensor array were amplified by the signal regulation board and converted to data by an A/D converter card with a 12 bits capacity. Then, these data were transmitted to the PC of the monitoring center via an IEEE 488 bus. To avoid the problem of re-pollution, the vacuum pump was connected to the outlet of the chamber housing the sensor array. The rotameter was used to maintain a constant flow of gas.

**Figure 3:**
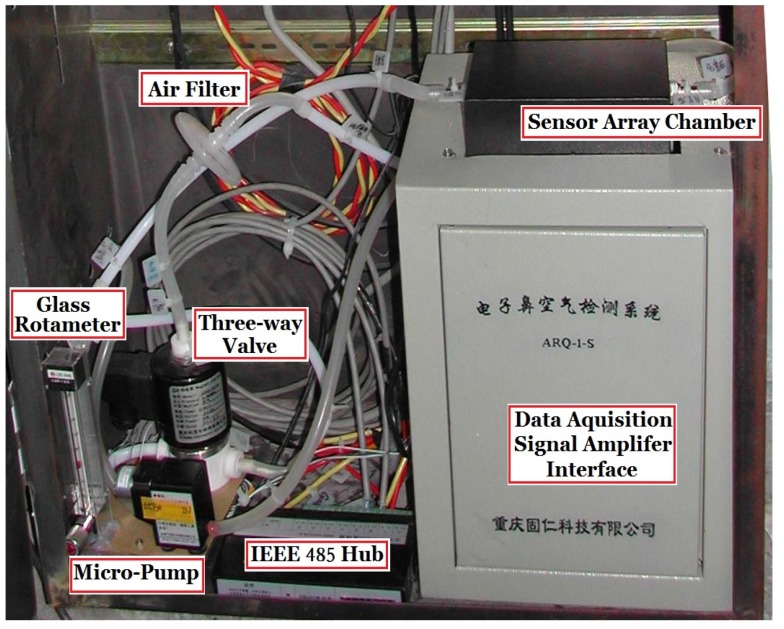
Photo of the e-nose system.

**Figure 4:**
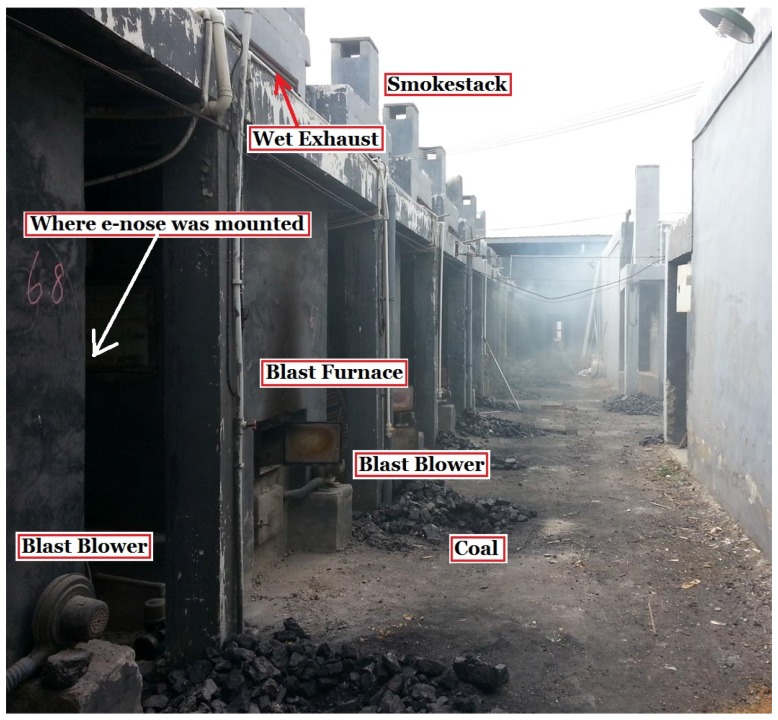
Photo of the tobacco curing barn.

Photos of the designed e-nose system and experimental site (a tobacco curing barn in the countryside) are shown in Figure 3 and Figure 4, respectively.

The whole e-nose was put beside the tobacco barn, and a micro pump was used to pump gas into the chamber from the barn, as shown in Figure 2.

**Figure 5:**
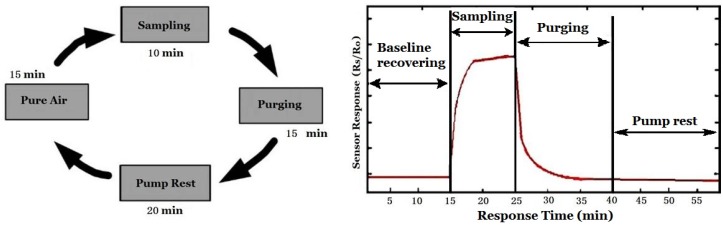
Smell data acquisition cycle and corresponding sensor response cycle.

Since the distance between the bulk curing-barn and the monitoring center PC ranges was several tens of meters, the IEEE 485 bus was used for remote data transmission. The operating process of the e-nose is as follows. The three-way valve was first switched to the outlet of the air filter so purified air was pumped to the sensor array chamber by the vacuum pump for 15 min. During this period, the gas sensors worked in baseline status. Then, the three-way valve was switched to the vent of the curing-barn and the gas, which contained the smells to be measured, was pumped to the sensor array chamber for 10 min. After that, the purified air was pumped into the sensor array chamber for 15 min to purge the sensors. Finally, it took 20 min for the vacuum pump to rest and one cycle was finished as shown in Figure 5 where the response of a gas sensor during the cycle is also given. The same process was repeated during the whole tobacco curing process. The repeated cycles lasted 7 days since the whole curing process lasts 7 days. Each hour, there is 1 sampling period (10 min), and 20 samples are collected during this 10-minute period. Since it needs 7 days to finish a whole curing process, almost 7 (days) × 24 (h) × 20 (samples) = 3360 samples are collected. All the samples are concatenated together to make a data sequence (curve).

### 3.2. Data Analysis—Strong Background Interference Existing at Outdoor Environment

The interference to an outdoor e-nose lies mainly in variations of temperature, humidity, atmospheric pressure and background smell.

#### 3.2.1. Interference from Temperature, Humidity and Atmospheric Pressure

**Figure 6:**
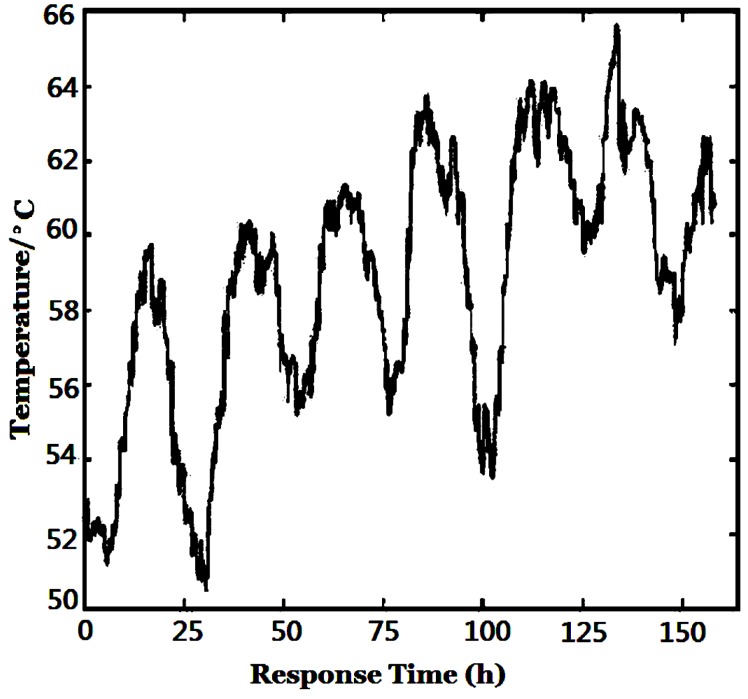
Variation of environment temperature.

**Figure 7:**
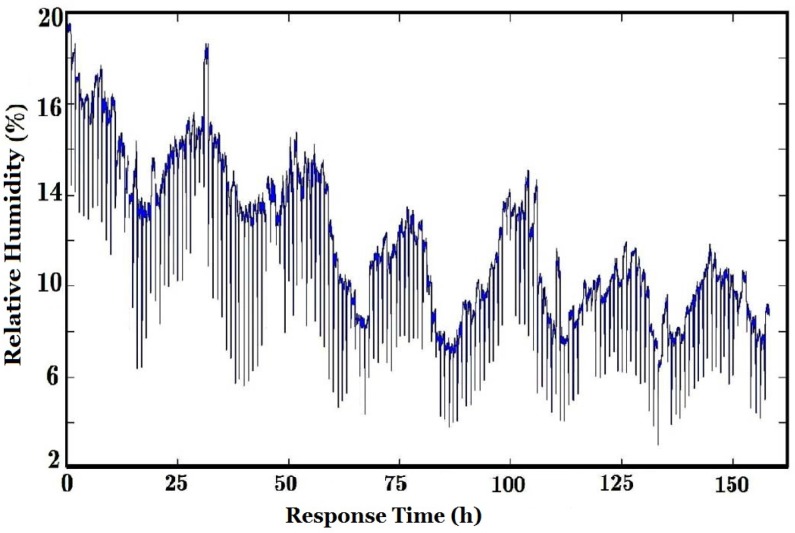
Variation of humidity level.

**Figure 8:**
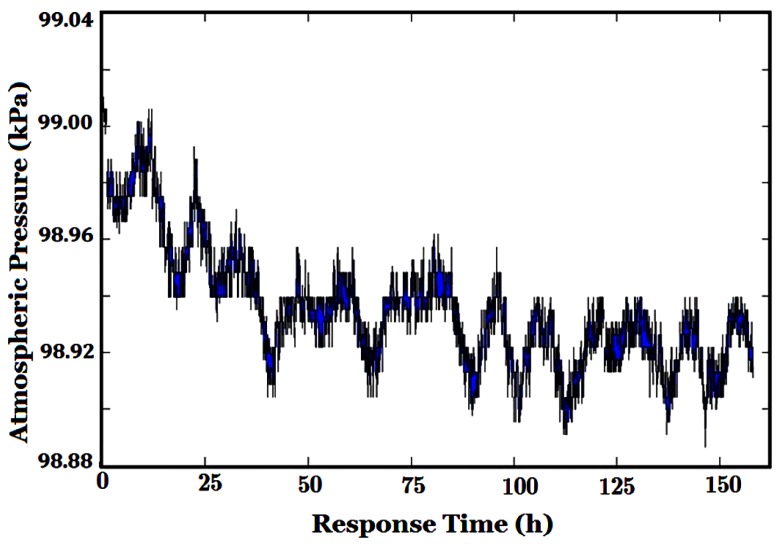
Variation of atmospheric pressure.

The gas sensors used in our e-nose are very susceptive to temperature and humidity (T/H). Their baselines vary greatly with T/H, even if only carrier gas/purified air appears [21]. The actual temperature, humidity and atmospheric pressure of the environment collected by corresponding sensors in the sensor chamber are shown in Figure 6, Figure 7 and Figure 8, respectively. The sensor array chamber is made of stainless steel with a layer of Teflon coating and it is not heated, so the temperature in the chamber shown in Figure 6 was affected both by the temperature of the gas from the barn and the air outdoors. Since the outdoor temperature difference between daytime and nighttime was more than 10 °C (the seven peaks and seven valleys correspond to 7 days’ daytime and nighttime, respectively), obviously, the influence of environmental T/H/P may not be ignored in a whole tobacco curing process.

The humidity level during the whole curing process is shown in Figure 7. To get the purified air, a set of filters such as activated carbon, molecular sieves and some canisters were used to filter out CO, SO_2_ and water, do the level of humidity was low at the purging stage. However, the humidity level in the chamber dynamically changed during the whole curing process due to the water released from tobacco.

#### 3.2.2. Interference of Environmental Smells on the Sensor Array

**Figure 9:**
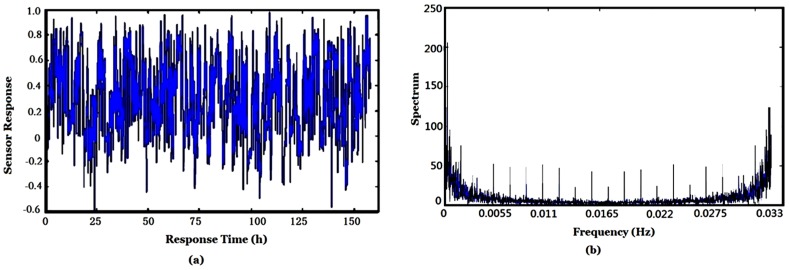
Response (**a**) and its spectrum (**b**) of e-nose sensor TGS813.

Due to the cross sensitivity of e-nose gas sensors, they are susceptible to many smells. In the scenario of current application, the interference comes mainly from pollutant gases, such as SO_2_, CO and nitrogen oxides, *etc*. which were produced by the burning coal. The TGS813 (FIGARO, Osaka, Japan) is one sensor of the e-nose sensor array. The on-site collected response of the TGS813 and corresponding amplitude of its Fourier transform are shown in Figure 9 where those points of sensor purging, bump resting and baseline are all omitted. To reduce the influence of environmental factors, each point in the curve was obtained by subtracting the baseline and divided by the difference of maximum and minimum of sensor response. The sampling frequency of the data acquisition is set to *f_s_* = 1/30 Hz = 0.033 Hz. Though the response of only one sensor is shown here, the responses of the other eight sensors are similar.

There are 20 intervals (peaks) in Figure 9b and it is easy to calculate the corresponding frequency, *i.e*., *f_s_*/20 = (1/30)/20 = 1/600 (Hz), so the corresponding time is 600 s (10 min). It corresponds to the sampling period of e-nose in Figure 5, and can be seen obviously in Figure 7.

### 3.3. Background Interference Suppression Procedure in an E-Nose

To control background interference in an e-nose, a method of separating environment-related interference factors under strong background interference was proposed in this paper. The steps of the algorithm are as follows:

(1) Pre-processing (including baseline removal, low-pass filtering and standardization);

(2) Principal component analysis (PCA);

(3) Independent component analysis (ICA) with the first several PCA components as inputs of ICA, removing those ICA output components which are strongly related to environmental interferences, then the ICA component which is more related to the true smell signal, may be obtained;

(4) By re-filtering the above obtained ICA component, a useful signal with environmental and background interferences suppressed in some extent is obtained.

In the above analysis, some *a priori* information, such as the time of coal feeding (which corresponds to the time the pseudo-periodical interference gas is produced), ambient temperature, humidity and atmospheric pressure information (an approximately periodical change of temperature and humidity is induced by the difference between daytime and nighttime) as well as the experts’ knowledge on tobacco smell, may be used.

#### 3.3.1. Pre-Processing

This includes removing the sensor baseline, low-pass filtering and data standardization:

(a) Baseline Removal and Normalization

A typical gas sensor response curve is shown in Figure 5. It comprises the stages of baseline recovery, sampling, purging and pump rest. To lessen the influence of environment changes (such as temperature, humidity and atmospheric pressure) on gas sensors, Equation (1) is used in the standardization:

(1) $$x_{i}=\frac{s_{i}-\mu_{i}}{\sigma_{i}}$$

where *s_i_* is the response (the voltage of the sensor, which is from a bleeder circuit [21]) of the *i*th sensor, *μ_i_* is the baseline, *σ_i_* is the standard deviation, *x_i_* is the output after standardization. In this way, the influence of ambient factors is expected to be reduced by some degree.

(b) Low-Pass Filtering

**Figure 10:**
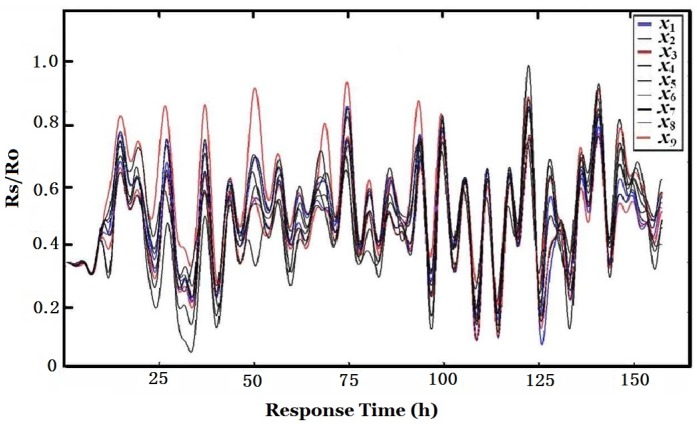
Low-pass filtered responses of the nine gas sensors.

The low-pass filter is used to filter out the interference of white noise. The main parameters of a low-pass filter include 3 dB bandwidth, cut-off frequency, type of filter, *etc*. By Fourier transform, the spectrum features and inherent interference frequencies may be found. For example, the frequency component corresponding to the 10 min sampling period may be found from Figure 9b, and the frequency bandwidth of the low-pass filter was selected to be able to filter out these periodical interferences. The low-pass filter is a three order low-pass filter. The cut-off frequency of this low-pass filter was set to 0.02 *f_s_*. The cut-off frequency of this low-pass filter should not be too low, otherwise, the independency of signal source will be destroyed and a bad ICA result may occur. The filtered responses of the array of nine sensors are shown in Figure 10.

It is shown in Figure 10 that the peaks in these curves are very consistent though the amplitudes of the gas sensors are different. Furthermore, it is found that the appearance time of these peaks is the very time coal was fed to the furnace, which hints that these peaks were produced by the coal smoke entering the curing barn. Since each time when coal was fed, a large amount of pollution gases (such as SO_2_, CO, *etc*.), which could be easily smelled by human noses, was produced due to incomplete burning. These pollutants were poured into the curing barn via its wet exhaust because it is close to smokestacks. Though each of the e-nose sensors was designed to be sensitive to some specific kinds of gases, they had extremely strong responses to these smokes due to their immense intensity while true tobacco smell was overwhelmed by these interfering gases. Low-pass filters with different cut-off frequencies were used, but the true signal of tobacco smell still could not be extracted since the true signal is in the same frequency band as that of smoke, environmental temperature, humidity and atmospheric pressure interferences. Next, the PCA and ICA are used to deal with the data.

#### 3.3.2. PCA Model and ICA Model

A 12-dimension response was obtained from the array of 12 sensors. While the PCA was used to reduce dimension and denoise, the environment related factors were found out by checking the PCA load coefficients.

**Figure 11:**
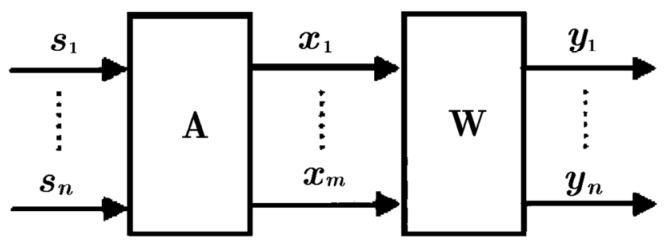
ICA model.

ICA is a useful method for blind source separation. It can separate statistically independent sources effectively so long as at most one source is of Gaussian distribution, as shown in Figure 11.

Assume there are *n* independent source signals *s*_1_(*t*), *s*_2_(*t*),…, *s_n_*(*t*) with their discrete forms *s*_1_, *s*_2_,…, *s_n_*, respectively; *x*_1_, *x*_2_,…,*x_m_* are the *m* arguments observed, the purpose of ICA is to estimate the *n* independent source through the *m* measured arguments. The ICA model is given by:

(2) X=AS

where **X** = (*x*_1_, *x*_2_,…,*x_m_*)^T^, **S** = (*s*_1_, *s*_2_,…, *s_n_*)^T^, **A** = (*a_ij_*), 1 ≤ *i* ≤ *m*; 1 ≤ *j* ≤ *n*. Both **A** and **S** are unknown. The only *a priori* information is that each component of **S** is statistically independent and at most one *s_i_*(*t*) is of Gaussian distribution. The target of ICA is to estimate the separation matrix **W**, which is the inverse of **A**, and further to get $\hat{S}$, the optimal estimation of **S**, with the components of $\hat{S}$ are independent as much as possible:

(3) $$\hat{S}=WX$$

The Fast ICA algorithm is a widely used algorithm and was adopted here [22]; it is based on the principle of maximized non-Gaussian feature. It searches out the non-Gaussian feature maximum of **WX** by iteration with the negative entropy approximation as target function.

Herein the actual application scenario is as follows: *m* = 2, *n* = 2 and *x*_1_, *x*_2_,…,*x_m_* are the output components of PCA (PCA1, PCA2), respectively; *s*_1_ (with ICA1 expressing its estimation) represents the source which is introduced by various gas smells; *s*_2_ denotes the other source (with ICA2 expressing its estimation) incurred by those environmental factors other than gas smells, such as temperature, humidity and atmospheric pressure.

After pre-processing in the way given in Section 3.1, the outputs of the 12 sensors *x*_1_, *x*_2_, …, *x*_12_ were sent to PCA. The eigenvalues, accumulated contribution rate and coefficients of the first two PCA components *y*_1_, *y*_2_ (denoted by PCA1 and PCA, respectively) are given in Table 2 and Table 3. From Table 2, it can be found that the accumulated contribution rate of the first two components of PCA (*i.e*., PCA1, PCA2) reached 89.3%, so it is reasonable to use these two components as features of the sensor array.

**Figure 12:**
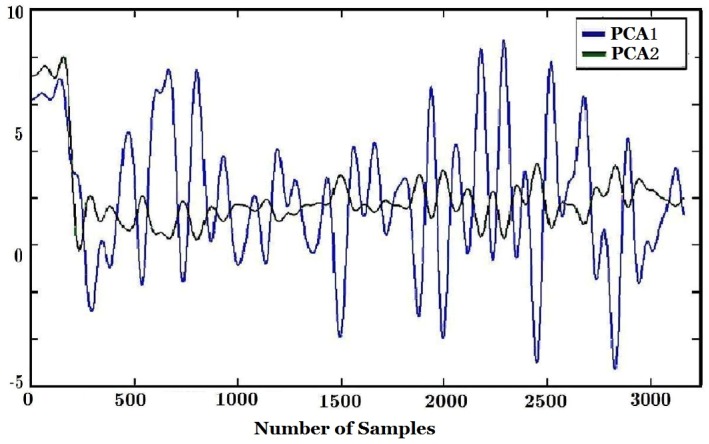
Curves of the first and second components of PCA.

From Table 3, it can be seen that the *t_y_* values of PCA2 for atmospheric pressure, temperature and humidity are 0.5962, 0.5390, 0.5381, respectively, and they are far bigger than those for other factors. This means that PCA2 mainly reflects the influence of environmental factors while PCA1 reflects the smell function of smell. The first two components of PCA are shown in Figure 12.

**Figure 13:**
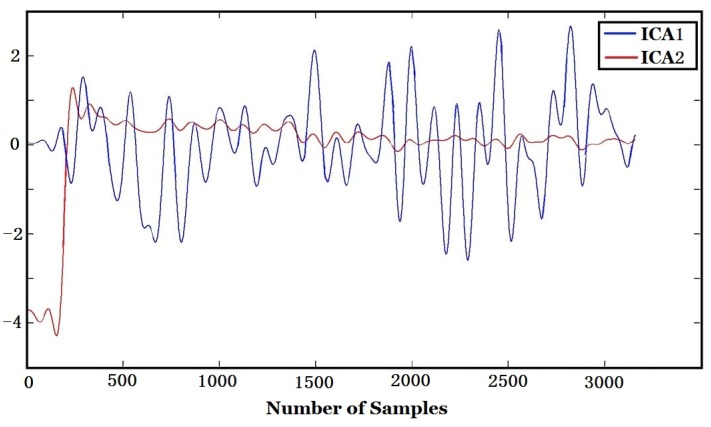
Two outputs (ICA1, ICA2) of ICA.

To further compensate the influence of environmental factors, ICA was used after PCA. The Fast ICA algorithm for ICA was adopted. The outputs of ICA, *i.e*., ICA1 and ICA2 ($\hat{S}$ in Equation (3)) are shown in Figure 13.

#### 3.3.3. Second Low-Filtering

Although ICA1 may be approximately regarded as being removed of the influence of environmental atmospheric pressure, temperature and humidity, there still exists (pseudo-periodical) interference from burning coal pollution. This has been confirmed by analyzing the response curves of sensors in Figure 10, the consistency between the peaks of ICA1 curve and the time of coal feeding. Since the independency of various gas smell sources cannot be guaranteed, the true tobacco smell cannot be obtained by ICA only. To restrain the interference from burning coal (particularly from the early burning stage) and other noises, the second low-pass filtering (re-filtering) was used to filter the output of ICA. The cut-off frequency of this low-pass filter was set to be lower than the corresponding frequency of the pseudo periods and the interference from burning coal smoke was suppressed to a certain extent.

It is worth noting that two low-pass filtering passes were adopted. The first low-pass filtering is before PCA, which is for white noise suppression; whereas the second low-pass filtering is after ICA, which is for removing the burning coal interference. The cut-off frequency of these two filters is different. The cut-off frequency of the first filtering is set far bigger than that of the second filtering, otherwise, environmental interference sources could not be separated correctly by ICA since too small a cut-off frequency destroys independencies among sources.

**Figure 14:**
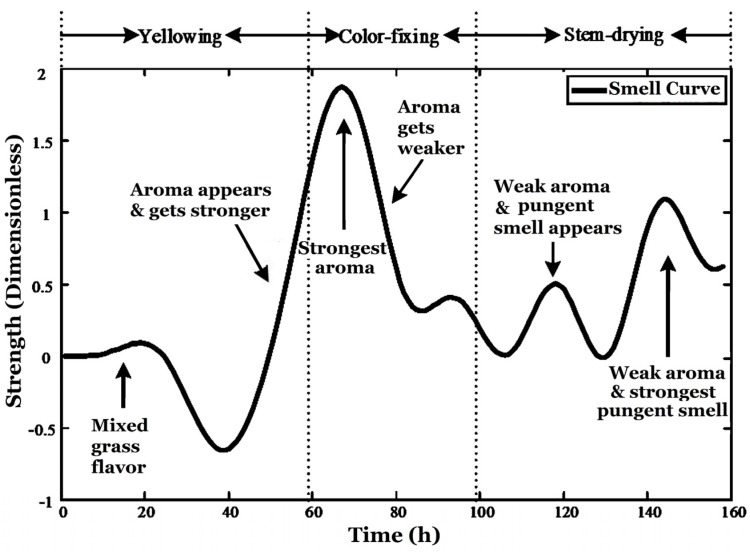
ICA1 after low-pass re-filtering (more related to tobacco smell).

The smell curve (*i.e*., ICA1 after the second low-pass filtering) together with the curer’s evaluation on smells are shown in Figure 14. As there were strong interferences produced by the burning coal, some interference removal measures were taken. The interferences were not totally eliminated, but the curve shown in Figure 14 can match the experiences of the curer and some research results, it can be considered as a reference and approximation of the true smell variations.

## 4. Result-Tobacco Smell Curve

The whole flue-curing process can be divided into three stages, *i.e*., yellowing, color-fixing and stem-drying shown in Figure 14 based on the status of the tobacco. Meanwhile, a professional curer in the tobacco factory was assigned to sniff the smell every 3 h and his experience was used as an intelligent reference in our smell modelling. According to the experiences of the curer and [19], at the yellowing stage, the smell is mixed grass flavor and it reduces gradually with the tobacco color becoming more and more yellow. Meanwhile, it gradually sends out a mellow flavor (aroma) and the smell curve begins to go up. During the color-fixing stage, after the leaves become totally yellow, the aroma reduces. Then at the early stem-drying stage, the dehydration of tobacco is stronger, the smell becomes a little bit pungent and it goes away along with the decreasing amount of moisture. When the free moisture of tobacco is exhausted, the smell is gradually replaced by the inherent smell of tobacco. The pungent smell is stronger at the end of flue-curing process. This curve is mainly consistent with the curers’ olfactory perception, while it is only a qualitative reflection of the smell variation during the whole curing stage. From Figure 14, it can be found that the e-nose cannot differentiate between aromatic smell and pungent smell. Also there are some fluctuations of smell curve during the whole curing process and the exact mechanism has not been found yet.

## 5. Discussion

We used coefficient of multiple correlation to measure the effectiveness of interference suppression in our method.

### 5.1. Coefficient of Multiple Correlation (CMC)

CMC and its improved counterpart have been used in frequency or wavelet domain for pattern recognition of complex data set [23], elbow arthrosis and gait dynamic data analysis [24,25] *etc*. CMC is used to measure the correlation between variable *y* and other multiple variables *x*_1_, *x*_2_, …, *x_k_*. Bigger CMC means stronger correlation [26]. To quantify the validity of separating interferences by either PCA or ICA, we used CMC to measure the linear correlation between PCA/ICA component and the environmental factors. Bigger CMC means that the corresponding PCA/ICA component has stronger correlation with environmental factors.

The steps of CMC computation are as follows:

(1) Calculate $\hat{y}$, the regression of *y* to $x_{1},\;x_{2},\;...,\;x_{k}$:

(4) $$\hat{y}=\beta_{o}+\beta_{1}x_{1}+\;...+\;\beta_{k}x_{k}$$

(2) Calculate the CMC between *y* and $x_{1},\;x_{2},\;...,\;x_{k}$:

(5) $$R=\frac{\sum \left(y-\bar{y})(\hat{y}-\bar{y}\right)}{\sqrt{\sum {\left(y-\bar{y}\right)}^{2}\sum {\left(\hat{y}-\bar{y}\right)}^{2}}}$$

### 5.2. Data Experiment Using CMC

When computing the CMC of PCA, *y* should be the first component (PCA1) and second component (PCA2), respectively. Since only the correlation between those environmental factors (responses of atmospheric pressure, temperature and humidity sensors, *i.e*., *x*_10_, *x*_11_, *x*_12_) and PCA1/PCA2 is considered, only *β*_0_ and *x*_10_, *x*_11_, *x*_12_ appear in the right of Equation (4).

Similarly, when the CMC of ICA is calculated, *y* should be the first component (ICA1) and second component (ICA2), respectively, while only *β*_0_ and *x*_10_, *x*_11_, *x*_12_ appear in the right of Equation (4).

**Figure 15:**
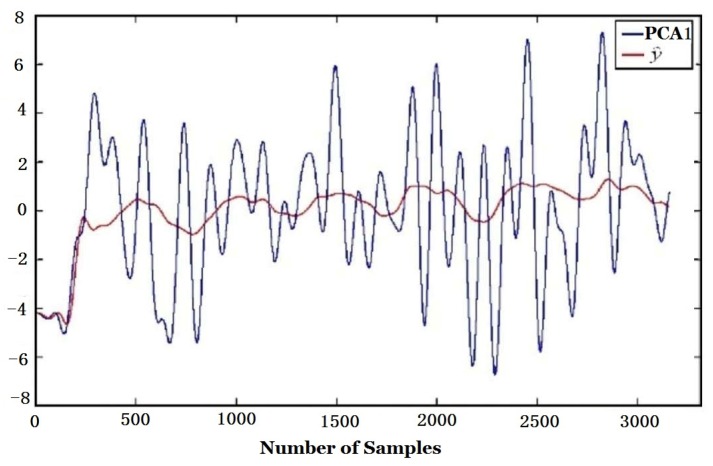
Regression of PCA1 to environmental factors.

**Figure 16:**
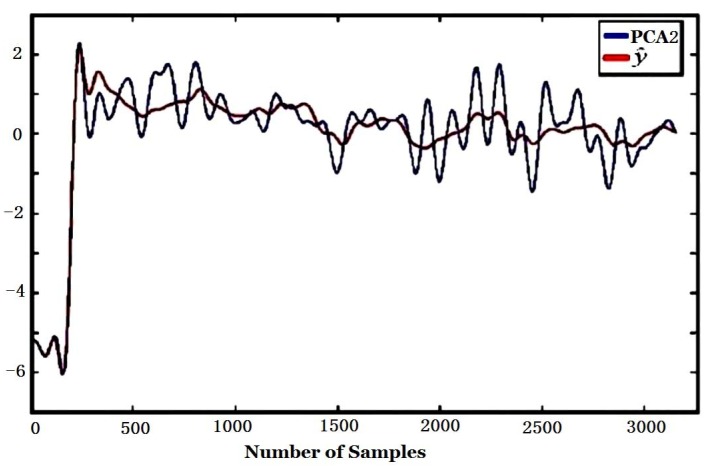
Regression of PCA2 to environmental factors.

The regression ($\hat{y}$ in Equation (4)) of the first and second components of PCA, *i.e*., PCA1 and PCA2 (*y* in Equation (5)) to environmental variables are given in Figure 15 and Figure 16, respectively. It is obvious that the regression of PCA2 to $\hat{y}$ is much better than that of PCA1. The regression coefficients calculated according to Equation (4) and the coefficient of multiple correlation *R* in Equation (5) are shown in Table 4. It is found that the CMC of PCA2 to environmental variables *R* is 0.9430, which is much bigger than that of PCA1, 0.4250. That means PCA2 is more related to environmental factors (atmospheric pressure, temperature, humidity).

**Figure 17:**
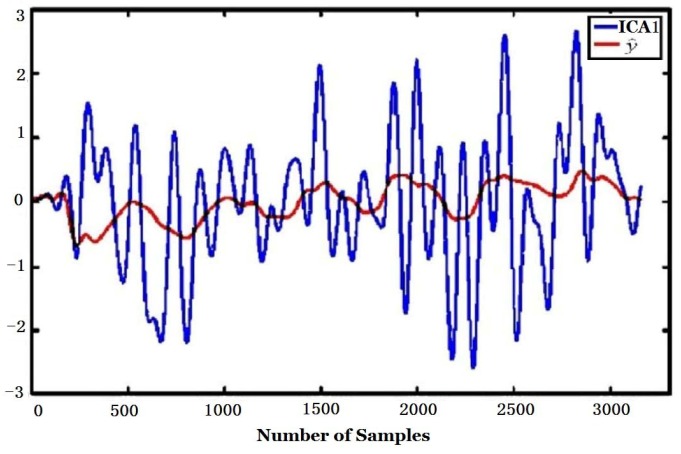
Regression of ICA1 to environmental variables.

**Figure 18:**
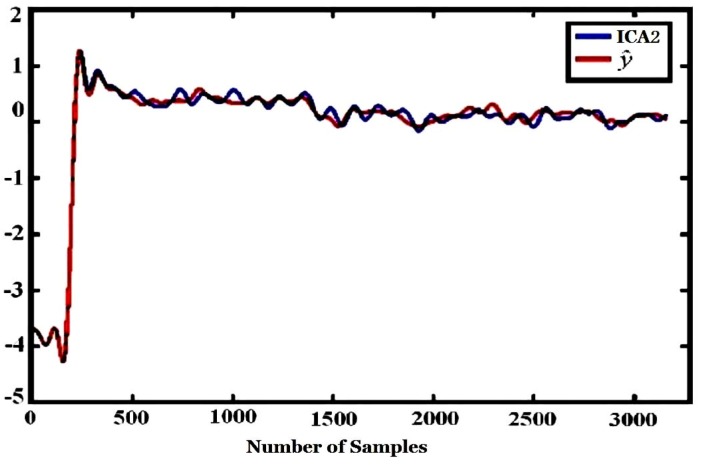
Regression of ICA2 to environmental variables.

The CMC and regression coefficients of ICA components to environmental variables computed according to Equations (4) and (5) are shown in Table 5. Figure 17 and Figure 18 are the regression curve of ICA1 and ICA2 to environmental variables, respectively. It can be found from Table 5 that since the CMC of ICA2 to environmental variables (R = 0.9967) is far bigger than that of ICA1 (R = 0.2763), ICA2 should be more related to environmental factors, and this can also be found from Figure 17 and Figure 18 where the regression curve of the former is worse than the latter. Comparing Table 4 with Table 5, it can be found that ICA2 is more related to environmental factors than PCA2 since the CMC of ICA2 (0.9967) is bigger than that of PCA2 (0.9430). This is also confirmed by the fact that the curve fitting in Figure 18 is better than that in Figure 16.

In contrast, the CMC of ICA1 to environmental factors is 0.2763 which is smaller than that of PCA1 (0.4250). That means environmental influences are much compensated for ICA1, which is obtained by ICA after PCA, than for PCA1. *i.e*., ICA1 is more eligible to represent true tobacco smell. Comparing ICA1 of Figure 17 with Figure 10, one can find that the peaks appearance times of both figures are the same and they are consistent with the coal feeding time.

## 6. Conclusions

In the outdoor environment, variations of atmospheric pressure, temperature and humidity as well as interferences are very large, and an e-nose sensor array is strongly affected. Based on the application scenario of the outdoor tobacco curing environment, a method of restraining/compensating strong interference for e-noses was proposed. The outputs of PCA were used as inputs of ICA. By comparing the CMC of PCA outputs and ICA outputs with environmental variables, the effectiveness of ICA after PCA in separating environmental influence is confirmed. Combined with *a priori* knowledge, two low-pass filtering steps were used to restrain noise and strong background interference (the smell produced by burning coal). The advantage of this method lies in effectively separating the sensor responses of smell from those of environmental interference factors. The environmental interferences were suppressed to a great degree. We hope, this method will not only be effective for e-nose sensors used in tobacco curing, but also will it be of reference value for e-noses used outdoors for other purposes. Since only the knowledge of coal feeding time (which was the time coal smog appeared) and human olfactory perception to both smog and tobacco smell was used as *a priori* information besides low-pass re-filtering, the interference from the background smell cannot be removed entirely. Here we only studied the off-line case. Next, we will study the on-line (real-time) application, the combination of deep learning, big data technique and non-linear method [27] to restrain background interference as much as possible. The data and code used in this paper may also be downloaded [28].

## Figures and Tables

**Table 1:** Sensors used in the E-nose. Many of the below sensors have responses to alcohol, but their responses to these key chemicals are different among suppliers, providing an increased amount of chemical information.

Sensor Type	No.	Related Sensitivity	Manufacturers
TGS826	1	Isobutane, ethanol, ammonia, hydrogen	FIGARO, Osaka, Japan
TGS813	2	Methane, propane, isobutane	FIGARO, Osaka, Japan
TGS822	3	Ethanol, organic solvents	FIGARO, Osaka, Japan
TGS 2600	4	Cigarette smoke	FIGARO, Osaka, Japan
TGS 2602	5	Volatile Organic Compounds (VOCs), ammonia, hydrogen sulfide	FIGARO, Osaka, Japan
MQ135	6	Ammonia, sulfide, BTEX, acetone, toluene, ethanol, carbon monoxide	Winsen, Zhengzhou, China
MQ138	7	Alcohols, ketones, aldehydes, aromatics, organic solvents	Winsen, Zhengzhou, China
WSP2111	8	Toluene, benzene, ethanol, acetone	Winsen, Zhengzhou, China
SP3S-AQ2	9	VOC, hydrogen, ethanol, methane, ammonia	FIS, Hyogo, Japan
MPX4100AP	10	Atmospheric pressure	Freescale, Austen, TX, USA
DS600	11	Temperature	MAXIM, Sunnyvale, CA, USA
HIH4000	12	Humidity	Honeywell, Morristown, NJ, USA

**Table 2:** PCA results.

No.	Eigenvalue	Contribution Rate	Accumulated Contribution Rate
1	8.4115	70.0955	70.0955
2	2.3058	19.2146	89.3101
3	0.5775	4.8127	94.1227
4	0.3452	2.8769	96.9996
5	0.2226	1.8553	98.8549
6	0.0600	0.5001	99.3550
7	0.0399	0.3324	99.6874
8	0.0177	0.1478	99.8352
9	0.0092	0.0768	99.9119
10	0.0048	0.0403	99.9523
11	0.0031	0.0258	99.9781
12	0.0026	0.0219	100.0000

**Table 3:** Coefficients of the first two PCA components.

No.	Inputs of PCA	PCA1	PCA2
1	*x*_1_	0.2990	−0.1481
2	*x*_2_	0.3211	0.0339
3	*x*_3_	0.3314	−0.0858
4	*x*_4_	0.3399	−0.0350
5	*x*_5_	0.3076	−0.0114
6	*x*_6_	0.3399	−0.0212
7	*x*_7_	0.3378	−0.0418
8	*x*_8_	0.3302	−0.1376
9	*x*_9_	0.3304	−0.1080
10	*x*_10_	0.1305	0.5962
11	*x*_11_	0.1430	0.5390
12	*x*_12_	0.0461	0.5381

**Table 4:** CMC and regression coefficients of the first two components of PCA to environmental variables *x*_10_, *x*_11_, *x*_12_.

Component of PCA	*β*_0_	*β*_10_	*β*_11_	*β*_12_	R
PCA1	−4.2102	−0.0541	0.1647	0.0021	0.4250
PCA2	−5.1636	0.0264	0.0274	0.1653	**0.9430**

**Table 5:** CMC and regression coefficients of ICA components to environmental variables.

Component of ICA	*β*_0_	*β*_10_	*β*_11_	*β*_12_	R
ICA1	−0.0437	0.0241	−0.0447	0.0433	0.2763
ICA2	3.6978	−0.0084	−0.0394	−0.0999	**0.9967**
